# A Novel de novo *KIF1A* Mutation in a Patient with Ataxia, Intellectual Disability and Mild Foot Deformity

**DOI:** 10.1007/s12311-022-01489-y

**Published:** 2022-10-13

**Authors:** Yuka Hama, Hidetoshi Date, Akiko Fujimoto, Ayano Matsui, Hiroyuki Ishiura, Jun Mitsui, Toshiyuki Yamamoto, Shoji Tsuji, Hidehiro Mizusawa, Yuji Takahashi

**Affiliations:** 1 Department of Neurology, National Center Hospital, National Center of Neurology and Psychiatry, 4-1-1 Ogawahigashimachi, Kodaira, Tokyo 187-8551 Japan; 2 Department of Orthopedics, National Center of Neurology and Psychiatry, National Center Hospital, Kodaira, Japan; 3 Department of Neurology, Graduate School of Medicine, The University of Tokyo, Tokyo, Japan

**Keywords:** *KIF1A*, Ataxia, Foot deformity, Whole-exome sequencing, Intellectual disability

## Abstract

Early-onset ataxias are often difficult to diagnose due to the genetic and phenotypic heterogeneity of patients. Whole exome sequencing (WES) is a powerful method for determining causative mutations of early-onset ataxias. We report a case in which a novel de novo *KIF1A* mutation was identified in a patient with ataxia, intellectual disability and mild foot deformity.

A patient presented with sporadic forms of ataxia with mild foot deformity, intellectual disability, peripheral neuropathy, pyramidal signs, and orthostatic hypotension. WES was used to identify a novel de novo mutation in *KIF1A*, a known causative gene of neurodegeneration and spasticity with or without cerebellar atrophy or cortical visual impairment syndrome (NESCAVS).

We report a novel phenotype of NESCAVS that is associated with a novel de novo missense mutation in *KIF1A*, which provides valuable information for the diagnosis of NESCAVS even in the era of WES. Early rehabilitation of patients with NESCAVS may prevent symptom worsening and improve the disease course.

**Supplementary Information:**

The online version contains supplementary material available at 10.1007/s12311-022-01489-y.

## Introduction

Early-onset ataxias are often challenging to diagnose due to the genetic and phenotypic heterogeneity of patients, and lack of specific clinical disease indicators. Identifying specific clinical features of ataxias may greatly improve the differential diagnosis of the diseases when mutational screening is negative for major ataxias including those involving triplet repeat expansion mutations. Whole exome sequencing (WES) is a powerful tool that is useful for identifying causative mutations [1]. However, it is important to determine whether mutations identified via WES actually explain disease phenotypes. Therefore, the identification of neural or extra-neural findings of patients with particular gene mutations have the potential to improve the WES data–based clinical diagnosis of ataxias. Here, we report the use of WES to identify a novel *KIF1A* mutation in a patient with ataxia, intellectual disability, and mild foot deformity.

## Case Report

A 25-year-old woman presented to our hospital with intellectual disability and severe gait disturbance. Her familial history revealed no consanguinity. Further, her father, mother, and two brothers were neurologically normal. The patient was developmentally normal until the age of 1.5 years, at which time her gait became unstable, and she experienced daily falls. When she was 2 and 3 years old, the patient used no more than two- and three-word sentences, respectively. After she entered elementary school, the patient was unable to perform at grade level, and was identified as having mental retardation. Gait disturbance slowly progressed as follows: at the age of 15 years, crutches were needed; at the age of 22 years, the patient began using a wheelchair.

**Fig. 1:**
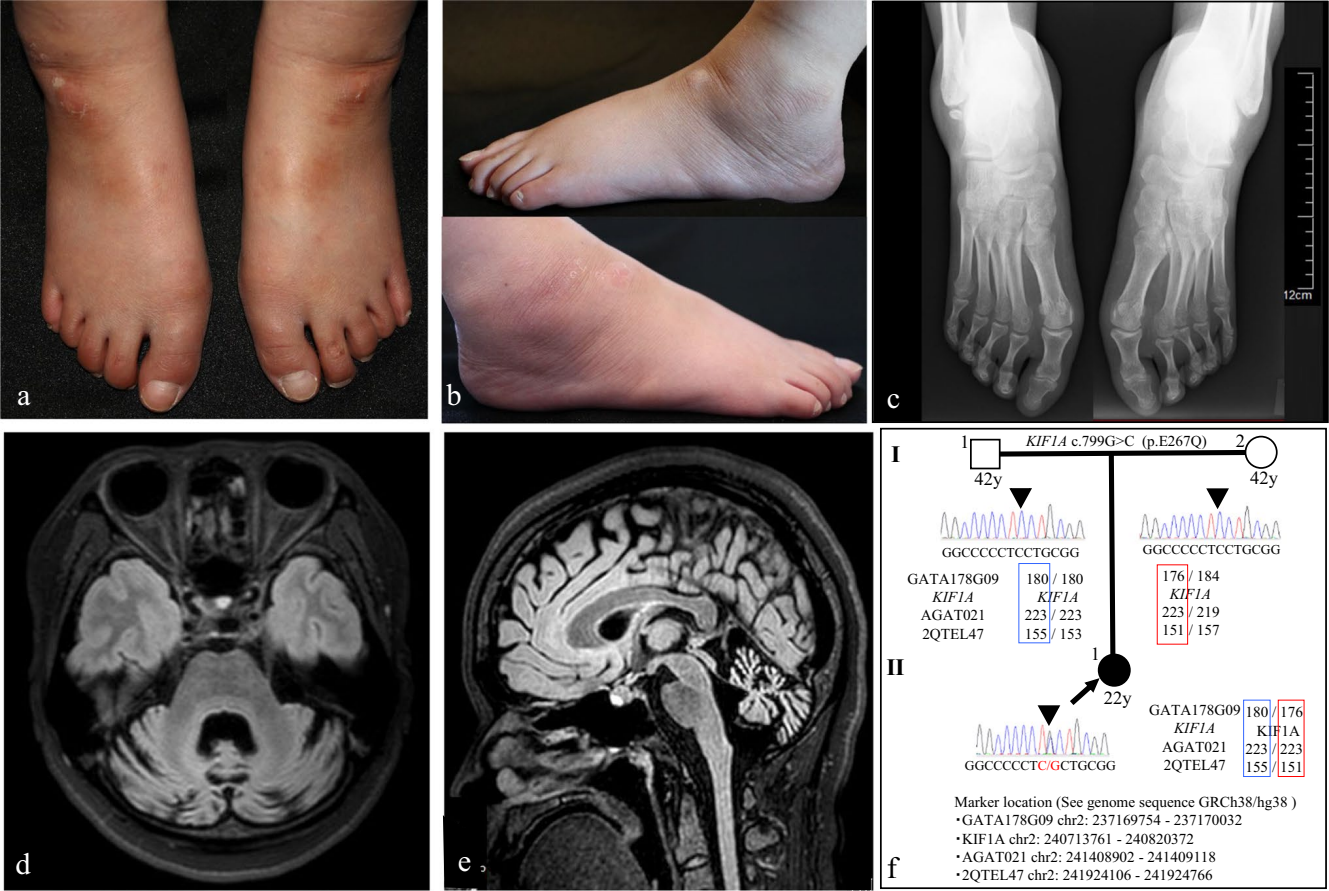
Patient symptoms and genetic features. The front (a) and lateral (b) views of the patient’s feet show that they are short and flat feet, with disproportionally narrow width in the anterior portion. The front view of the plain radiographs of the patient’s feet (c) showed hypoplastic calcaneus bones and arches. Axial (d) and sagittal (e) views of fluid-attenuated inverted recovery (FLAIR) MRI images of the brain revealing severe cerebellar atrophy and moderate dilatation of the fourth ventricle are shown. Whole exome sequencing analysis (WES) identified a novel heterozygous mutation in the *KIF1A* gene [c.799G > C (p.E267Q)]. Electropherograms of direct nucleotide sequence analyses are shown (f) on the left side of corresponding individuals. The arrow indicates the index patient and arrowheads indicate a mutation site. The mutation was not identified in the patient’s parents. Therefore, it was considered a de novo mutation based on the consistent parent-descendant relationship supported by the genotyping of three surrounding microsatellite markers (GATA178G09, AGAT021 and 2QTEL47)

A neurological examination performed at the age of 22 revealed severe cerebellar ataxia, mild muscle weakness of the lower limbs, a reduced upper-limb tendon reflex, an increased tendon reflex of the lower limbs without overt spasticity, bilateral positive Babinski and Chaddock signs, vibration sensation impairment at the ankles, orthostatic hypotension, and cognitive dysfunction. Her scale for the assessment and rating of ataxia (SARA), Mini-Mental State Exam scores were 18 and 21, respectively. Further, Wechsler Adult Intelligence Scale scores were 59, 50 and 51 for VIQ, PIQ and FIQ, respectively. Foot deformity (Fig. 1a, b) was noted, with the patient presenting with short, flat feet, that both were disproportionally narrow at the anterior portion with reference to relatively wide posterior portion, as compared to the feet of 2406 healthy Japanese people (Supplementary Fig. 1).

Results of biochemical analyses including the measurement of vitamin E, albumin, and alpha-fetoprotein levels, and a CSF examination were normal. X-ray examination of her foot revealed hypoplastic calcaneus bones and arches (Fig. 1c). A nerve conduction study showed that the patient had a decreased compound muscle action potential amplitude (4.2 mV), and a slow motor conduction velocity (39.6 m/s) in the tibial nerve. No sensory nerve action potential was observed in the sural nerve. An electromyogram showed poly-phasic motor unit potentials (MUPs) in the biceps brachii and high amplitude and poly-phasic MUPs and fibrillation potentials in tibialis anterior muscle. Magnetic resonance imaging of the brain revealed severe cerebellar atrophy and moderate dilatation of the fourth ventricle (Fig. 1d, e). Findings of 99 m Tc-ethyl cysteinate dimer single photon emission computed tomography revealed cerebellar hypoperfusion. Neither cerebral atrophy nor hypoperfusion was observed.

Her DNA sample was obtained and subjected to mutational analysis after the patient provided informed consent. Her parents assisted her upon obtaining a consent. Initial polymerase chain reaction-based fragment analysis for major types of spinocerebellar ataxia including Dentatorubral-pallidoluysian atrophy, Machado-Joseph disease/SCA3, and spinocerebellar ataxia types 1, 2, 6, 7, 8, 12, 17, and 31 were negative. WES revealed a novel heterozygous mutation in the *KIF1A* gene (c.799G > C [p.E267Q]). The mutation was determined to be located within the motor domain, a region known as a mutational hotspot. The sequences are highly conserved across species (Supplementary Fig. 2). The mutation was not identified within a 1261 sample, in-house, control dataset, Japanese Multi Omics Reference Panel (jMorp; ToMMo 4.7KJPN, https://jmorp.megabank.tohoku.ac.jp/201909/), nor was it identified in a The Genome Aggregation Database (gnomAD) (https://gnomad.broadinstitute.org/). Since the mutation was not identified in the patient’s parents, we concluded that it was a de novo mutation. A parent-descendant relationship was confirmed by genotyping three surrounding microsatellite markers (Fig. 1f), six single nucleotide polymorphisms (SNPs) in the vicinity of *KIF1A* gene (Supplementary Fig. 3), and 8 loci of triplet repeat diseases identified during initial screening (Supplementary Fig. 4). Based on these finding, the patient was diagnosed with neurodegeneration and spasticity with or without cerebellar atrophy or cortical visual impairment syndrome (NESCAVS, MIM [Mendelian Inheritance in Man] 614255).

Her symptoms gradually worsened, and her SARA score deteriorated from 18 to 22 throughout the following year, despite treatment with taltirelin hydrate and protirelin tartrate. Initiation of an intensive rehabilitation program tailored for ataxia based on a previous study [2] stabilized her symptoms and maintained her SARA score at 22 for 3 years.

## Discussion

We report a sporadic ataxia patient associated with a novel de novo missense mutation in *KIF1A* presenting with ataxia, intellectual disability, and mild foot deformity. NESCAVS is an early-childhood-onset neurodegenerative disorder characterized by a group of neurological features including developmental delay, visual symptoms, ataxia, spasticity and peripheral neuropathy [3]. NESCAVS is caused by a mutation in the *KIF1A* gene [3], a gene also implicated in spastic paraplegia 30 (SPG30, MIM 610,357) and neuropathy, hereditary sensory, type IIC (HSN2C, MIM 614,213). Although the patient described in this report did not exhibit visual symptoms, her complex phenotype matched neurological features of NESCAVS.

The identification of extra-neural symptoms and signs often provides clues needed to obtain correct diagnosis for neurological disorders. Patients with cerebellar ataxia, particularly those with disorders complicated with young-onset peripheral neuropathy, may present with foot deformities including pes cavus, clubfeet, and hammer toes [4–6]. The etiology of foot deformity remains elusive; however, various theories regarding the mechanistic details of foot deformity formation have been postulated [7]. These theories include abnormal bone and joint development, generalized ligamentous laxity of soft tissue, traumatic deformities, and neuromuscular imbalance [8]. KIF1A belongs to the kinesin 3 family. Kinesins are a large superfamily of molecular motor proteins, which move along microtubule filaments that drive intracellular transport and cell division. Skeletal deformity is one of the common clinical manifestations of disorders associated with kinesin mutations [8, 9]. KIF1 promotes chondrocyte maintenance during skeletal morphogenesis and regulates osteoclastic bone resorption [10, 11]. Notably, two patients with heterozygous missense mutations located withing the kinesin motor domain of *KIF1A*, c.902G>A (p.R307Q) and c.595G>A (p.G199R), showed clubfoot or joint contractures, respectively [12]. The mutation identified in our patient was also located in the kinesin motor domain suggesting the functional relevance to the previous reports, although the difference of phenotypes may be due to other genetic or environmental factors. In addition, one patient with a mutation c.5271dupC (p.S1758Qfs∗7) of *KIF1A* had equinus deformities [13]. Therefore, it is reasonable to suppose that the foot deformity observed in this case might be a clinical feature that is associated with a *KIF1A* mutation.

WES has become a major procedure to conduct the accurate molecular diagnosis. However, the problem is that once we detect novel variants by WES, it is sometimes difficult to decide whether the variants fully account for the phenotypes instead of mere variants with unknown significance (VUS). Reports with detailed clinical description are highly valuable in this context, because it would make phenotype matching of the patients with *KIF1A* variants with NESCAVS and final clinical diagnosis more accurate and feasible. Further accumulation of clinical evidence regarding foot deformity in KIF1A-associated diseases may improve the diagnosis and early awareness of NESCAVS.

Intensive rehabilitation has been reported to improve motor performance in degenerative cerebellar diseases [2]. Particularly, it has both short- and long-term effects on gait in patients with spinocerebellar ataxia [2]. The significance of rehabilitation was suggested in pediatric patients with *KIF1A* mutations manifesting as spastic paraplegia phenotypes, for whom a deterioration in mobility likely occurred due to progressive spasticity, muscle weakness, and the secondary development of severe contractures [14]. Our patient had stable SARA scores for 3 years following the initiation of intensive rehabilitation, an improvement to progressive worsening by 4 points throughout the previous year, suggesting its therapeutic effect on ataxia symptoms. Underlying mechanisms of therapy are presumed to be the activation of cerebellar neuronal functioning and related networks, and the prevention of secondary disabilities due to disuse. Intriguingly, in animal models, endurance training significantly increased kinesin levels in the sciatic nerves of male Wistar rats with diabetic neuropathy [15]. It might be worth investigating whether intensive rehabilitation in kinesin-associated diseases provides benefits via the alteration of kinesin levels throughout the nervous system.

In conclusion, we report a novel phenotype of NESCAVS that is associated with a novel de novo missense mutation in *KIF1A*. We would like to emphasize that the constellation of phenotypes in this patient provides valuable information for the diagnosis of NESCAVS even in the era of WES, particularly for the accuracy and feasibility of phenotype matching. Finally, early rehabilitation of patients with NESCAVS may prevent symptom worsening and improve the disease course.

### Supplementary Information

Below is the link to the electronic supplementary material.

Supplementary file1 (PDF 3429 KB)

Supplementary file2 (PDF 45 KB)

Supplementary file3 (PDF 47 KB)

Supplementary file4 (PDF 35 KB)
