# Correction: A Novel de novo KIF1A Mutation in a Patient with Ataxia, Intellectual Disability and Mild Foot Deformity

**DOI:** 10.1007/s12311-022-01490-5

**Published:** 2022-11-02

**Authors:** Yuka Hama, Hidetoshi Date, Akiko Fujimoto, Ayano Matsui, Hiroyuki Ishiura, Jun Mitsui, Toshiyuki Yamamoto, Shoji Tsuji, Hidehiro Mizusawa, Yuji Takahashi

**Affiliations:** 1 Department of Neurology, National Center Hospital, National Center of Neurology and Psychiatry, 4-1-1 Ogawahigashimachi, Kodaira, Tokyo, 187-8551 Japan; 2 Department of Orthopedics, National Center of Neurology and Psychiatry, National Center Hospital, Kodaira, Japan; 3 Department of Neurology, Graduate School of Medicine, The University of Tokyo, Tokyo, Japan

**Correction: The Cerebellum**

**https://doi.org/10.1007/s12311-022-01489-y**

The original version of this article unfortunately contained an error.

The author, Dr. Hiroyuki Ishiura, was not assigned to its affiliation, which is Affiliation 3 to read “Department of Neurology, Graduate School of Medicine, The University of Tokyo, Tokyo, Japan”.

The original article has been corrected.

