# Supplementary material for: A Novel de novo KIF1A Mutation in a Patient with Ataxia, Intellectual Disability and Mild Foot Deformity
Source: Cerebellum. 2022 Oct 13;22(6):1308–11. doi: 10.1007/s12311-022-01489-y (PMC10657280; doi:10.1007/s12311-022-01489-y)
Supplement: Supplementary file 1 — Supplementary file1 (PDF 3429 KB) [file 12311_2022_1489_MOESM1_ESM.pdf]

Supplementary Figure 1

Data of 2406 men and women (ages 19-96 years), mainly Japanese over 18 years old, who can walk independently without significant deformation of both lower limbs

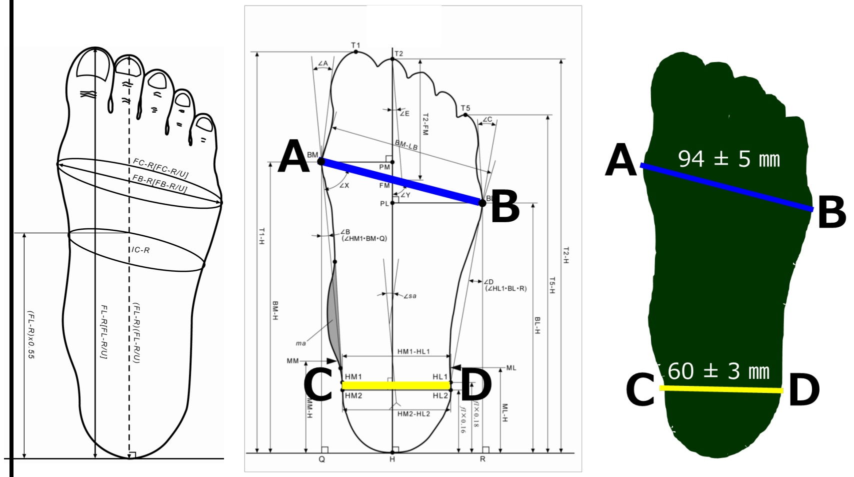

From the data of "2009 Japan Leather Industry Association Foot Size Measurement Business Report"

Our case

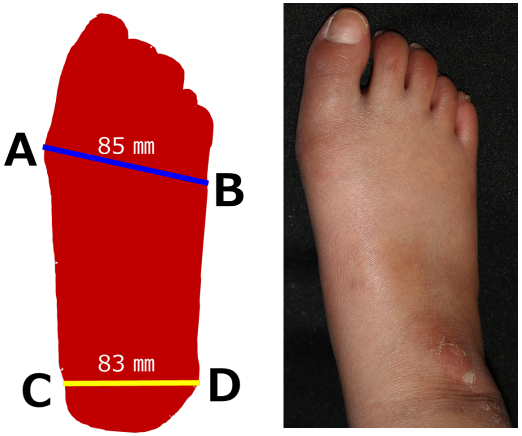

Source: 2009 report on the foot size measurement project of the Japan Leather Industry Federation.
