# Supplementary material for: A Novel de novo KIF1A Mutation in a Patient with Ataxia, Intellectual Disability and Mild Foot Deformity
Source: Cerebellum. 2022 Oct 13;22(6):1308–11. doi: 10.1007/s12311-022-01489-y (PMC10657280; doi:10.1007/s12311-022-01489-y)
Supplement: Supplementary file 2 — Supplementary file2 (PDF 45 KB) [file 12311_2022_1489_MOESM2_ESM.pdf]

Supplementary Figure 2

p.E267Q

▼

Homo sapiens	GTRLKEGANIN	NP_001230937.1
Pan troglodytes	GTRLKEGANIN	XP_016806380.2
Macaca mulatta	GTRLKEGANIN	XP_028687158.1
Mustela putorius furo	GTRLKEGANIN	XP_012907321.2
Sus scrofa	GTRLKEGANIN	XP_020930520.1
Mesocricetus auratus	GTRLKEGANIN	XP_012973907.1
Rattus norvegicus	GTRLKEGANIN	XP_038940678.1
Mus musculus	GTRLKEGANIN	NP_001395854.1
Gallus gallus	GTRLKEGANIN	XP_040534902.1
Xenopus tropicalis	GTRLKEGANIN	XP_031758411.1
