# Supplementary material for: A Novel de novo KIF1A Mutation in a Patient with Ataxia, Intellectual Disability and Mild Foot Deformity
Source: Cerebellum. 2022 Oct 13;22(6):1308–11. doi: 10.1007/s12311-022-01489-y (PMC10657280; doi:10.1007/s12311-022-01489-y)
Supplement: Supplementary file 3 — Supplementary file3 (PDF 47 KB) [file 12311_2022_1489_MOESM3_ESM.pdf]

Supplementary Figure 3

**Reference SNP (rs) number**	**Position** (GRCh38/hg38)	**Patient**	**Father**	**Mother**
rs1553637932 (the mutation in the proband)	chr2:240783109	G/**C**	G/G	G/G
rs2288748	chr2:240784010	A/A	A/A	A/G
rs891991042	chr2:240784437	C/T	C/T	C/T
rs768776074	chr2:240784440	C/T	C/T	C/T
No rs number	chr2:240784443	C/T	C/T	C/T
No rs number	chr2:240784621	C/G	C/G	C/G
No rs number	chr2:240784679	G/C	G/C	G/C
