# Supplementary figures and images for: A Novel de novo KIF1A Mutation in a Patient with Ataxia, Intellectual Disability and Mild Foot Deformity
Source: Cerebellum. 2022 Oct 13;22(6):1308–11. doi: 10.1007/s12311-022-01489-y (PMC10657280; doi:10.1007/s12311-022-01489-y)

Supplementary Figure 4

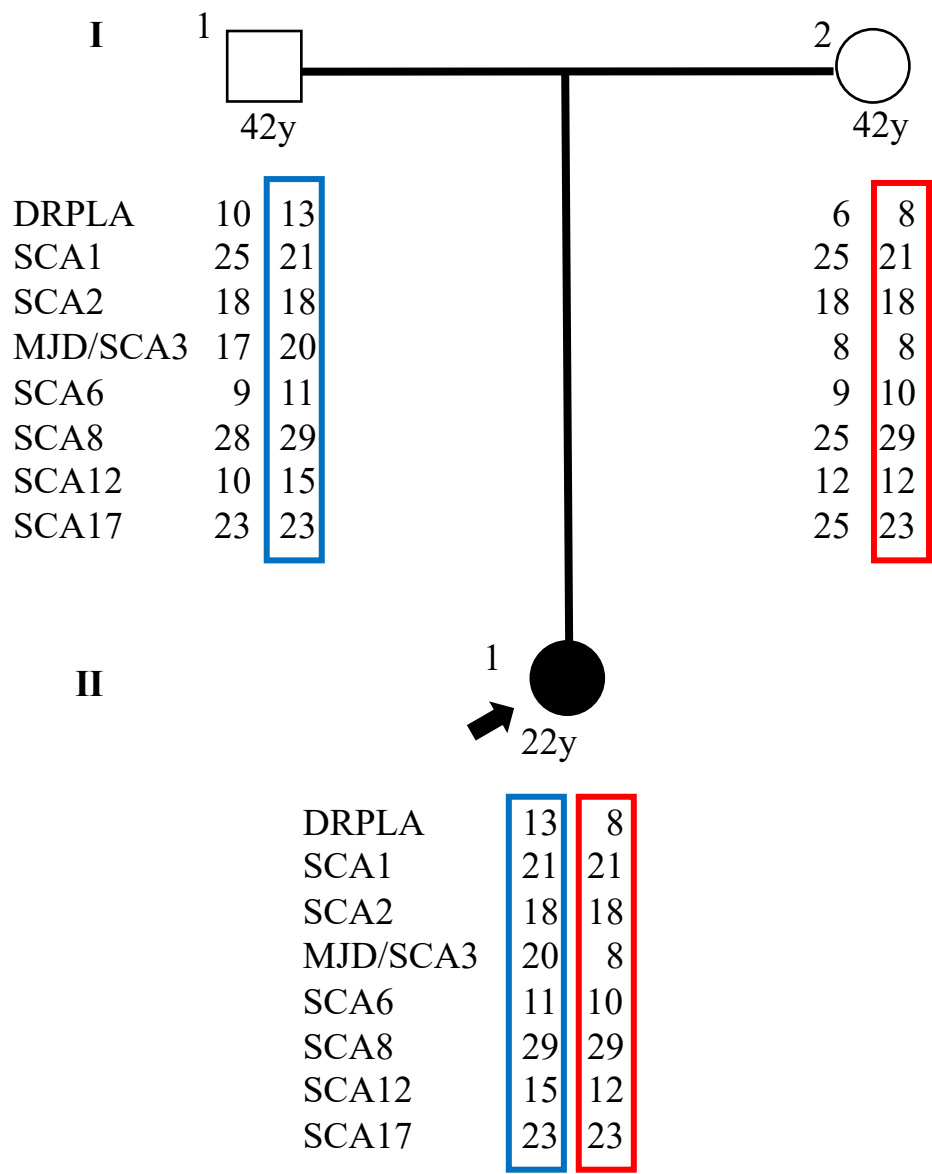

Supplement: Supplementary file 4 — Supplementary file4 (PDF 35 KB) [file 12311_2022_1489_MOESM4_ESM.pdf]
